# Effect of supplementation with rumen-protected choline and green tea extract on production performance of transition Karan Fries cows

**DOI:** 10.14202/vetworld.2020.489-494

**Published:** 2020-03-14

**Authors:** Parag Acharya, S. S. Lathwal, Pawan Singh, Neela Madhav Patnaik, Baisakhi Moharana

**Affiliations:** 1 Division of Livestock Production and Management, National Dairy Research Institute, Karnal, Haryana, India; 2 Division of Dairy Extension, National Dairy Research Institute, Karnal, Haryana, India; 3 Division of Pharmacology, Council of Scientific and Industrial Research-Central Drug Research Institute, Lucknow, Uttar Pradesh, India

**Keywords:** green tea extract, milk fat, milk yield, production, rumen-protected choline, transition Karan Fries cows

## Abstract

**Aim:**

The main objective of this study was to estimate the effect of supplementation of rumen-protected choline (RPC) and green tea extract (GTE) on production parameters in transition Karan Fries (KF) cows.

**Materials and Methods:**

The present experiment was carried out on 32 pregnant KF cows. In the control group, cows were fed basal diet. In T1, each cow was fed RPC (55 g/day), in T2 – GTE (3 g/d), and in T3– RPC + GTE (55+3) g/day along with basal diet. The duration of the experiment was 30 days before calving to 60 days after parturition.

**Results:**

Feeding of both RPC and GTE significantly increased milk yield (p≤0.01), 4% fat corrected milk yield (p≤0.01), milk fat (p≤0.01), and total solid content (p≤0.05) than control. There was no significant difference (p≥0.05) in milk protein, lactose, and solids not fat (SNF) content among the groups.

**Conclusion:**

Supplementing RPC and GTE in combination improved milk yield and fat content of the milk without altering protein, lactose content of the milk in transition KF cows.

## Introduction

The transition period, roughly stretching from 3 weeks before to 3 weeks after parturition [1], is a most critical physiological period for high-yielding dairy cows, and is characterized by a high incidence of metabolic, infectious, and reproductive disorders [2,3]. Increased demand of energy and nutrients for the synthesis of colostrum and milk along with decreased feed intake forced the transition cows to undergo negative energy balance (NEB). NEB along with severe oxidative stress leads to mobilization of fat in terms of non-esterified fatty acids and subsequent accumulation of beta-hydroxybutyric acid in blood [3].

Choline, chemically beta-hydroxyethyltrimethylammonium ion, is synthesized endogenously and an integral part of biological tissues as cytoskeleton components [4]. Rumen-protected choline (RPC), protected from rumen microbial degradation, acts as a methyl donor, hence leaving a larger supply of methionine for milk production [5]. In numerous studies, researchers have observed a tendency for higher milk yield due to the alleviation of NEB, decreased risk of fatty liver and ketosis, reproductive problems, and oxidative stress [2,3,6].

The beneficial properties of green tea are due to the abundant polyphenolic compounds (“catechins”), and these catechins include catechin (C), (2)-epicatechin (EC), (2)-epigallocatechin (EGC), (2)-EC-3-gallate, and (2)-EGC-3-gallate [7]. Green tea has anti-tumor effects such as induction of apoptosis, anti-angiogenesis, antioxidant, and anti-inflammatory effects [8,9]. There have been scanty reports of green tea, reducing metabolic and inflammatory stress around parturition in dairy animals [10], hence improving the production status.

This study aimed to explore the effect of supplementation of RPC and green tea extract (GTE) on production parameters in transition cows.

## Materials and Methods

### Ethical approval

Ethical permission was granted for the experiment by the Institutional Animal Ethical Committee of Indian Council of Agricultural Research-NDRI constituted as per article 13 of the CPCSEA rules, laid down by the Government of India (Regd no-1705/GO/al/13 CPCSEA).

### Experiment

This study was conducted in the cattle shed unit of National Dairy Research Institute (NDRI), Karnal, India. The RPC was purchased from Kemin Animal Nutrition, India, which was prepared by spray freeze-drying technology, in the form of encapsulation with fatty acids. The GTE was purchased from Sarthak Herbs, Karnal.

Thirty-two pregnant dairy cows were selected from the herd with most probable production ability (most probable producing ability [MPPA]) of around 4000 L milk production, in their second to fourth lactation stage and maintained at cattle yard, NDRI, Karnal. The MPPA or expected producing ability was computed on the basis of formula as follows:


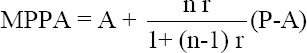


Where A = Population mean

n = Total number of animals

r = Repeatability of lactation milk record

P = Milk yield in previous lactation

They were fed basal diet constituting of concentrate mixture, green fodder (sorghum, maize, oats, and sugargraze), and dry roughage (wheat straw), as per NRC, 2001 [11].

The experimental groups were divided as follows:

- Control (C) – basal diet without supplementation
- Treatment 1 (T1) – basal diet with RPC (55 g/day)
- Treatment 2 (T2) – basal diet with GTE (3 g/day)
- Treatment 3 (T3) – basal diet with RPC (55 g/day) + GTE (3 g/day).

The experiment was started around 37 days before the expected date of parturition and given an adaption period of 7 days. The total duration of the experiment was 90 days. The treatment was given from 30 days before calving to 60 days after calving. All the calves belong to the same season, although few days’ variations were there in terms of birth date of the calves. The birth weight of calves was not recorded.

### Observations recorded during the study

#### Production status

Milk yield, 4% fat corrected milk (FCM), and energy corrected milk (ECM)

Animals were machine milked thrice a day, i.e., 05.00 h, 11.30 h, and 18.00 h up to 70 days postpartum. Milk samples were collected at each milking and proportionately pooled to represent milk samples of that animal at a weekly interval. Daily milk yield was recorded for each individual animal.

Calculation of FCM

For the conversion of whole milk into 4% FCM, the following equation was adopted [12].

4% FCM = 0.4 M + 15* F*MY/100

here, M = Milk yield (kg)

F = Milk fat (kg)

Calculation of ECM

For the conversion of whole milk into ECM, the following equation was adopted

ECM =MY * (kg/d) * (38.3*fat (g/kg) + 24.2 * protein (g/kg) + 16.54 * lactose (g/kg) + 20.7)/3140

MY = Milk yield (kg)

Milk composition

Representative milk samples were analyzed for their composition (fat, protein, SNF, and lactose) using a pre-calibrated milk analyzer (Lacto Star, FUNKE GERBER, Article No 3510, Berlin). The total solids in milk were calculated as follows:

Total Solid = Milk fat % + Milk SNF %

### Statistical analysis

Data were represented as mean±standard error. One-way analysis of variance (GraphPad Prism 5.01 software; San Diego, California, USA) was adapted to find out the significant difference between groups and day of treatments and to interpret the effect of dietary treatment on the various parameters.

## Results and Discussion

There was no significant difference between the groups in terms of fortnightly body weights of Karan Fries (KF) cows and dry matter intake (data not provided).

### Effect of RPC and GTE on Milk yield, 4% FCM yield, and ECM

All the treatments were able to increase the milk yield significantly (p≥0.01) from control although, cows fed both RPC and GTE (T3) had a significant (p≥0.01) higher milk yield than control and individual treatments by 10^th^ week (Table-1). In T2, 4% FCM also increased statistically significantly (p≤0.01) than control, though no significant difference (p≥0.05) was obtained between T1 and T2 (Table-2). However, in T3, overall 4% FCM is significantly higher (p≤0.01) than control, T1 and T2. Weekly average ECM ranged from 12.37±0.65 to 12.13±0.38 in control, 13.60±0.62-14.01±0.41 in T1, 13.63±0.44-13.17±0.62 in T2, and 15.28±0.54-15.27±0.28 in T3 (Table-3). Supplementation of both RPC and GTE had significant (p≤0.01) increase in ECM than other treatments and control in 7^th^, 8^th^, and 10^th^ weeks.

**Table-1:** Effect of RPC and GTE supplementation on milk yield (kg/d) of KF cows.

Weeks	C	T1	T2	T3
1^st^*	12.50^a^±0.49	13.21^a^±0.34	13.48^ab^±0.54	14.75^b^±0.42
2^nd^*	12.55^a^±0.51	13.68^ab^±0.59	13.97^ab^±0.59	14.77^b^±0.23
3^rd^*	12.54^a^±0.49	14.79^b^±0.59	14.57^b^±0.53	15.47^b^±0.23
4^th^**	12.83^a^±0.49	15.02^b^±0.48	14.81^b^±0.36	15.65^b^±0.13
5^th^**	13.11^a^±0.65	15.04^b^±0.37	14.98^b^±0.37	15.79^b^±0.74
6^th^**	13.16^a^±0.48	15.39^b^±0.39	15.39^b^±0.31	15.79^b^±0.06
7^th^**	13.55^a^±0.38	15.80^b^±0.25	15.57^b^±0.19	15.92^b^±0.06
8^th^**	13.34^a^±0.43	15.53^b^±0.29	15.40^b^±0.18	15.63^b^±0.14
9^th^*	13.28^a^±0.43	14.64^b^±0.48	14.34^ab^±0.33	14.88^b^±0.38
10^th^**	12.77^a^±0.49	14.43^bc^±0.29	13.86^ab^±0.64	15.34^c^±0.18
OM±SE**	12.96^a^±0.12	14.75^b^±0.26	14.64^b^±0.23	15.40^c^±0.14

Mean bearing different superscripts between the treatments differ significantly. Data represented as mean±SE (**p<0.01, *p<0.05). RPC=Rumen-protected choline, GTE=Green tea extract, KF=Karan Fries, SE=Standard error

**Table-2:** Effect of RPC and GTE supplementation on fat corrected milk yield (kg/d) of KF cows.

Weeks	C	T1	T2	T3
1^st^*	12.71^a^±0.70	14.00^ab^±0.68	13.89^ab^±0.51	15.82^b^±0.71
2^nd^*	12.61^a^±0.62	14.22^ab^±0. 57	14.21^ab^±0.93	15.97^b^±0.57
3^rd^**	12.52^a^±0.55	15.17^b^±0.62	14.87^b^±0.53	16.44^b^±0.45
4^th^**	12.92^a^±0.47	15.17^bc^±0.49	14.96^b^±0.48	16.39^c^±0.19
5^th^**	13.03^a^±0.63	15.33^b^±0.41	15.06^b^±0.53	16.41^b^±0.14
6^th^**	13.07^a^±0.63	15.62^b^±0.40	15.52^b^±0.29	16.45^b^±0.16
7^th^**	13.28^a^±0.42	15.37^b^±0.29	15.03^b^±0.27	16.39^c^±0.12
8^th^**	12.89^a^±0.47	15.21^b^±0.39	15.17^b^±0.16	16.13^b^±0.17
9^th^*	12.99^a^±0.45	14.74^b^±0.63	14.29^ab^±0.37	15.39^b^±0.41
10^th^**	12.61^a^±0.45	14.49^bc^±0.45	13.88^ab^±0.64	15.83^c^±0.22
OM±SE**	12.86^a^±0.08	14.93^b^±0.17	14.69^b^±0.18	16.12^c^±0.11

Mean bearing different superscripts between the treatments differ significantly. Data represented as mean±SE (**p<0.01, *p<0.05). RPC=Rumen-protected choline, GTE=Green tea extract, KF=Karan Fries, SE=Standard error

**Table-3:** Effect of RPC and GTE supplementation on an energy corrected milk yield (kg/d) of KF cows.

Weeks	C	T1	T2	T3
1^st^*	12.37^a^±0.65	13.60^ab^±0.62	13.63^ab^±0.44	15.28^b^±0.54
2^nd^**	12.24^a^±0.58	13.84^ab^±0.59	13.85^ab^±0.89	15.65^b^±0.39
3^rd^**	12.05^a^±0.53	14.67^b^±0.68	14.46^b^±0.58	16.07^b^±0.43
4^th^**	12.42^a^±0.38	14.69^b^±0.45	14.55^b^±0.45	15.62^b^±0.27
5^th^**	12.49^a^±0.65	14.69^b^±0.47	14.67^b^±0.49	16.03^b^±0.15
6^th^**	12.58^a^±0.69	14.90^b^±0.43	15.14^b^±0.36	15.9^b^±0.18
7^th^**	12.69^a^±0.44	15.01^b^±0.32	14.63^bc^±0.32	15.96^c^±0.19
8^th^**	12.43^a^±0.44	14.72^b^±0.32	14.49^b^±0.16	15.59^c^±0.18
9^th^*	12.50^a^±0.39	14.27^b^±0.70	13.59^ab^±0.40	14.91^b^±0.39
10^th^**	12.13^a^±0.38	14.01^bc^±0.41	13.17^ab^±0.62	15.27^c^±0.28
OM±SE**	12.39^a^±0.06	14.44^b^±0.15	14.22^b^±0.19	15.63^c^±0.12

Mean bearing different superscripts between the treatments differ significantly. Data represented as mean±SE (**p<0.01, *p<0.05). RPC=Rumen-protected choline, GTE=Green tea extract, KF=Karan Fries, SE=Standard error

Although the choline requirement of dairy cows is still unknown [11], higher choline availability (by feeding RPC) can have a favorable effect on milk production. At the time of parturition, there is a high need for methylated compounds and their availability through diet is limited. RPC acted as a methyl group donor and it provided more methyl groups in the RPC supplemented groups [13]. Furthermore, choline has a sparing effect on methionine since methionine can replace choline as the methyl group source and that choline can reduce methionine utilization for methylation reaction. The reduction of methyl group demand from the methionine pool may therefore offer production benefits to dairy ruminants [14]. This reason might explain the significant increase in milk yield in RPC supplemented group. In the present study, milk yield had increased significantly (p≤0.05) in GTE supplemented group as compared to control. However, Winkler *et al*. [10] did not find any significant difference in milk yield when supplemented 3.1 g of plant product consisting of GTE (95%) and curcuma extract (5%) to Holstein Friesians cows, 3 weeks before and 9 weeks after parturition. The significant increase in milk yield in GTE provided group might be due to the fact that, a potential reduction in inflammation and metabolic stress in the liver [10]. Inflammation around parturition is associated with increased heat production (fever) and production of acute-phase proteins in liver and immune system tissues [15]. Energy and amino acid required for that process may not available for milk production, which is the reason why inflammatory condition reduces milk production [16]. GTE, a potent hepatoprotective and anti-inflammatory agent [17,18], thus improved the milk production in the treated group. In T3 group, the average milk yield is significantly higher (p≤0.01) than control, T1 and T2. It might be due to the fact that, when RPC and GTE were combined together, they had produced a synergistic effect. Davidson *et al*. [6] found that multiparous cows fed RP-choline at 40 g/d had higher milk FCM and ECM yield than cows fed control. Winkler *et al*. [10] supplemented 0.175 g of plant product consisting of 95% green tea and 5% curcuma extract per kg DM of product and found that cows supplemented with polyphenols had an increased amount of ECM. Although, dry matter intake (data not included) did not differ significantly across the groups, it is likely that treatment of RPC and GTE led to an improvement of the utilization of energy for milk production, as a result of which, ECM in the treatment group increased. In our study, higher FCM in RPC and GTE supplementation group might be due to increased milk yield and milk fat percentage.

### Effect of RPC and GTE on milk composition

There was significantly (p≤0.01) higher milk fat percentage in T3 than control and other treatments (Table-4). The trend of significant (p≤0.05) increase in milk yield started from 3^rd^ week up to 10^th^ week in the T3 group. There was no significant difference among groups in milk protein concentration (Table-5) (but, the overall average milk protein percentage was significantly higher [p≤0.05] in T1 and T3 than control), milk SNF (Table-6) and lactose concentration (Table-7) in different weeks. Milk total solid percentage was significantly higher (p≤0.05) in T 1, T 2 and T 3 than control in 8^th^ and 9^th^ weeks of lactation (Table-8). The reason for higher milk solid concentration in the treatment group is due to higher fat content in the treatment groups.

**Table-4:** Effect of RPC and GTE supplementation on milk fat (percent) of KF cows.

Weeks	C	T1	T2	T3
1^st^*	4.34±0.05	4.34±0.05	4.33±0.05	4.35±0.08
2^nd^**	4.32±0.08	4.28±0.12	4.11±0.33	4.56±0.32
3^rd^**	3.99^a^±0.09	4.18^ab^±0.10	4.14^ab^±0.05	4.41^b^±0.13
4^th^**	4.05^a^±0.05	4.07^a^±0.03	4.06^a^±0.09	4.31^b^±0.05
5^th^**	3.96^a^±0.06	4.12^ab^±0.05	4.02^ab^±0.09	4.26^b^±0.05
6^th^**	3.94^a^±0.11	4.10^ab^±0.06	4.06^ab^±0.09	4.28^b^±0.05
7^th^**	3.86^a^±0.05	3.83^a^±0.15	3.78^a^±0.14	4.20^b^±0.04
8^th^**	3.78^a^±0.05	3.86^a^±0.09	3.90^a^±0.05	4.21^b^±0.05
9^th^*	3.85^a^±0.04	4.04^ab^±0.14	3.98^a^±0.05	4.23^b^±0.05
10^th^**	3.93^a^±0.06	4.03^a^±0.09	4.01^a^±0.05	4.21^b^±0.04
OM±SE**	3.95^a^±0.03	4.09^b^±0.05	4.03^ab^±0.04	4.32^c^±0.04

Mean bearing different superscripts between the treatments differ significantly. Data represented as mean±SE (**p<0.01, *p<0.05). RPC=Rumen-protected choline, GTE=Green tea extract, KF=Karan Fries, SE=Standard error

**Table-5:** Effect of RPC and GTE supplementation on milk protein (percent) of KF cows.

Weeks	C	T1	T2	T3
1^st^	3.26±0.09	3.34±0.14	3.43±0.10	3.36±0.14
2^nd^	3.23±0.10	3.33±0.13	3.30±0.07	3.30±0.12
3^rd^	3.14±0.11	3.21±0.17	3.29±0.10	3.31±0.11
4^th^	3.14±0.09	3.21±0.10	3.29±0.08	3.10±0.12
5^th^	3.06±0.11	3.14±0.16	3.31±0.09	3.33±0.12
6^th^	3.11±0.11	3.08±0.13	3.31±0.09	3.33±0.09
7^th^	3.03±0.14	3.28±0.13	3.21±0.09	3.35±0.09
8^th^	3.16±0.07	3.26±0.14	3.11±0.06	3.31±0.08
9^th^	3.18±0.13	3.26±0.09	3.08±0.14	3.30±0.07
10^th^	3.23±0.12	3.35±0.06	3.01±0.11	3.29±0.10
OM±SE*	3.15^a^±0.02	3.25^b^±0.03	3.23^ab^±0.04	3.29^b^±0.02

Mean bearing different superscripts between the treatments differ significantly. Data represented as mean±SE (**p<0.01, *p<0.05). RPC=Rumen-protected choline, GTE=Green tea extract, KF=Karan Fries, SE=Standard error

**Table-6:** Effect of RPC and GTE supplementation on milk SNF (percent) of KF cows.

Weeks	C	T1	T2	T3
1^st^	8.71±0.07	8.78±0.05	8.74±0.03	8.70±0.04
2^nd^	8.64±0.05	8.71±0.05	8.74±0.07	8.70±0.07
3^rd^	8.56±0.09	8.66±0.07	8.70±0.06	8.63±0.06
4^th^	8.66±0.05	8.61±0.06	8.66±0.06	8.73±0.04
5^th^	8.66±0.05	8.69±0.06	8.78±0.05	8.64±0.04
6^th^	8.61±0.04	8.60±0.04	8.66±0.06	8.66±0.07
7^th^	8.44±0.06	8.64±0.04	8.70±0.08	8.54±0.05
8^th^	8.55±0.06	8.65±0.03	8.59±0.03	8.65±0.05
9^th^	8.54±0.07	8.55±0.09	8.68±0.09	8.56±0.07
10^th^	8.60±0.07	8.54±0.07	8.48±0.07	8.50±0.09
OM±SE	8.59±0.02	8.64±0.02	8.67±0.03	8.63±0.08

Data represented as mean±SE. RPC=Rumen-protected choline, GTE=Green tea extract, KF=Karan Fries, SE=Standard error

**Table-7:** Effect of RPC and GTE supplementation on milk Lactose (percent) of KF cows.

Weeks	C	T1	T2	T3
1^st^	4.34±0.05	4.38±0.05	4.33±0.05	4.35±0.08
2^nd^	4.33±0.08	4.33±0.05	4.31±0.07	4.33±0.05
3^rd^	4.30±0.07	4.31±0.11	4.31±0.06	4.29±0.07
4^th^	4.34±0.05	4.34±0.06	4.29±0.04	4.26±0.06
5^th^	4.30±0.06	4.26±0.07	4.28±0.05	4.29±0.06
6^th^	4.29±0.05	4.26±0.08	4.30±0.07	4.27±0.07
7^th^	4.28±0.06	4.28±0.08	4.29±0.08	4.21±0.06
8^th^	4.20±0.07	4.16±0.09	4.15±0.07	4.14±0.06
9^th^	4.19±0.09	4.21±0.07	4.15±0.09	4.23±0.19
10^th^	4.15±0.09	4.23±0.08	4.24±0.08	4.24±0.08
OM±SE	4.27±0.02	4.26±0.03	4.26±0.02	4.26±0.02

Data represented as mean±SE. RPC=Rumen-protected choline, GTE=Green tea extract, KF=Karan Fries, SE=Standard error

**Table-8:** Effect of RPC and GTE supplementation on milk TS (percent) of KF cows.

Weeks	C	T1	T2	T3
1^st^	12.85±0.28	13.16±0.26	12.99±0.27	13.19±0.31
2^nd^	12.69±0.25	12.99±0.11	12.85±0.31	13.26±0.32
3^rd^	12.55±0.15	12.84±0.14	12.84±0.08	13.04±0.14
4^th^	12.71±0.08	12.68±0.05	12.73±0.08	13.05±0.06
5^th^	12.63±0.08	12.81±0.09	12.79±0.12	12.90±0.06
6^th^	12.55±0.12	12.70±0.08	12.73±0.13	12.94±0.09
7^th^	12.29±0.07	12.46±0.07	12.48±0.05	12.74±0.08
8^th^**	12.32^a^±0.05	12.51^b^±0.08	12.49^b^±0.04	12.86^c^±0.04
9^th^*	12.39^a^±0.06	12.59^ab^±0.13	12.65^ab^±0.09	12.79^b^±0.08
10^th^	12.53±0.09	12.56±0.14	12.49±0.09	12.71±0.13
OM±SE*	12.55^a^±0.06	12.73^ab^±0.07	12.70^ab^±0.06	12.95^bc^±0.06

Mean bearing different superscripts between the treatments differ significantly. Data represented as mean±SE (**p<0.01, *p<0.05). RPC=Rumen-protected choline, GTE=Green tea extract, KF=Karan Fries, SE=Standard error

Pandurang [19] found a significant increase in milk fat percentage (p≤0.05) in RPC treated group. However, Leiva *et al*. [20] did not notice any significant difference in milk fat percentage in RPC supplemented group. As choline (as phosphatidylcholine) plays a role in fatty acid transport in blood, it is speculated that more lipoprotein triglycerides may have been available to the mammary gland for incorporation into milk fat. Either it might be due to the fact that RPC channelizes lipid absorption and transport, thereby encouraging the fatty acid transport from adipose to mammary tissue and helped in milk fat synthesis. Winkler *et al*. [10] also found no significant difference in milk fat percentage in green tea and curcuma extract supplemented group, though daily fat yield (kg/d) increased significantly (p≤0.05) in the treated group. However, in T3, significantly higher (p≤0.05) milk fat percentage was found as compared to C, T1 and T2. The probable reason might be the lipotropic nature of choline, which made more lipoprotein triglyceride available to the mammary gland for milk fat synthesis.

Several mechanisms have been suggested for the positive effect of dietary RPC on milk protein synthesis: (1) A sparing of methyl groups (and thus Met as the methyl group donor) needed for choline synthesis; (2) RPC may serve as a methyl group donor for remethylation of homocysteine through its metabolite betaine; and (3) betaine may also spare Met by responding as methyl group donor in some metabolic processes [21]. This would leave a larger supply of Met for milk protein synthesis and would be especially effective in cows fed diets with a low Met passage to the small intestine [22]. Protein concentration increased significantly (p≤0.05) in T3 as compared to C, T1 and T2 might be due to the sparing effect of choline, which provided more methyl group for milk protein synthesis. However, Winkler *et al*. [10] found no significant difference in milk protein percentage in green tea and curcuma extract supplemented group, though daily milk protein yield (kg/d) increased significantly (p≤0.05) in the treated group.

No significant difference was found across the groups for milk SNF percentage. The findings in the present study are in agreement with those of Sheikh *et al*. [23]. In contrary to this, Pandurang [19] found a significant increase in milk SNF percentage (p≤0.05) in RPC treated cows. Likewise, SNF percentage, no significant difference was found across the groups for milk lactose concentration. Our result is in agreement with the previous authors [19,21,23]. Winkler *et al*. [10] also found no significant difference in milk lactose percentage in green tea and curcuma extract supplemented group. Hence, treatments did not have any deleterious effect on milk composition for which it can be supplemented in the diet to have increased milk yield.

## Conclusion

Supplementation of RPC and GTE enhanced milk production and had a positive and desirable effect on milk quality with enhanced milk fat and total solid content. This improvement might be the result of supplementing GTE, which alleviate oxidative challenge faced by the animals at the critical transition phase.

## Authors’ Contributions

This study was conducted as part of the Ph.D. research program at NDRI, Karnal. SSL, PS, and PA involved in the genesis of the hypothesis. PA has conducted the whole experiment. PA, BM, and NMP were involved in data analysis and proofreading of the manuscript. All the authors were participated in the drafting and revision of the manuscript and approved the final version.
